# Social support and poverty alleviation among asylum-seekers and refugees in Wales

**DOI:** 10.1093/eurpub/ckac130.170

**Published:** 2022-10-25

**Authors:** M Wells, H Seage

**Affiliations:** Psychology, Cardiff Metropolitan University, Cardiff, UK

## Abstract

**Background:**

Wales is working toward being a Nation of Sanctuary for asylum-seekers and refugees and has resettled between 6,000 and 10,000 refugees since 2001. Uprooted social support networks during the period of asylum-seeking process predispose this population to poverty. The aim of this research was to explore the role of social support among asylum-seekers and refugees in alleviating poverty and its adverse impact on their health and wellbeing. Understanding the importance of social support in promoting the health and wellbeing of asylum-seekers and refugees is a critical factor for informing public health interventions and policies to improve outcomes for tackling poverty among this population in Wales.

**Methods:**

A qualitative research method, based on social support framework, was adopted. Semi-structured interviews were utilised to collect data from a purposive sample of 8 participants. All the participants were recruited through a refugee support organisation in Cardiff, Wales and were interviewed in the community hub of this organisation. The interviews were digitally recorded, transcribed and analysed using Interpretative Phenomenological Analysis (IPA).

**Results:**

Results show two formal (government, charities) and three informal (family, friends, and peers) sources of social support as the potential pathways that alleviate the impact of poverty on the health and wellbeing of asylum-seekers and refugees. These sources provide access to language courses, education, employment, housing, food, and sense of belonging in the new communities of resettlement.

**Conclusions:**

Enhancing access to formal and informal sources of social support is of vital importance to tackling the pernicious impact of poverty on the health and wellbeing of asylum-seekers and refugees. Providing social support for this population should be given uttermost consideration for public health interventions and policy decisions in an effort to protect and promote their health and wellbeing.

**Key messages:**

- Public health interventions and policies should consider access to social support resources by asylum-seekers and refugees.
- Access to, and quality of, social support resources will alleviate the impact of poverty and improve the health and wellbeing of asylum-seekers and refugees in the host country.

